# Primary care physician characteristics associated with cancer screening: a retrospective cohort study in Ontario, Canada

**DOI:** 10.1002/cam4.358

**Published:** 2014-11-27

**Authors:** Aisha K Lofters, Ryan Ng, Rebecca Lobb

**Affiliations:** 1 Department of Family and Community Medicine, University of TorontoToronto, Ontario, Canada; 2 Department of Family and Community Medicine, St. Michael's HospitalToronto, Ontario, Canada; 3 Centre for Research on Inner City Health, The Keenan Research Centre in the Li Ka Shing Knowledge Institute of St. Michael's HospitalToronto, Ontario, Canada; 4 Institute for Clinical Evaluative SciencesToronto, Ontario, Canada; 5 Division of Public Health Sciences, Department of Surgery and Alvin J. Siteman Cancer Center, Washington University School of MedicineSt. Louis, Missouri

**Keywords:** Cancer screening, immigrant health, primary care, socioeconomic status

## Abstract

Primary care physicians can serve as both facilitators and barriers to cancer screening, particularly for under-screened groups such as immigrant patients. The objective of this study was to inform physician-targeted interventions by identifying primary care physician characteristics associated with cancer screening for their eligible patients, for their eligible immigrant patients, and for foreign-trained physicians, for their eligible immigrant patients from the same world region. A population-based retrospective cohort study was performed, looking back 3 years from 31 December 2010. The study was performed in urban primary care practices in Ontario, Canada's largest province. A total of 6303 physicians serving 1,156,627 women eligible for breast cancer screening, 2,730,380 women eligible for cervical screening, and 2,260,569 patients eligible for colorectal screening participated. Appropriate breast screening was defined as at least one mammogram in the previous 2 years, appropriate cervical screening was defined as at least one Pap test in the previous 3 years, and appropriate colorectal screening as at least one fecal occult blood test in the previous 2 years or at least one colonoscopy or barium enema in the previous 10 years. Just fewer than 40% of physicians were female, and 26.1% were foreign trained. In multivariable analyses, physicians who attended medical schools in the Caribbean/Latin America, the Middle East/North Africa, South Asia, and Western Europe were less likely to screen their patients than Canadian graduates. South Asian-trained physicians were significantly less likely to screen South Asian women for cervical cancer than other foreign-trained physicians who were seeing region-congruent patients (adjusted odds ratio: 0.56 [95% confidence interval 0.32–0.98] versus physicians from the USA, Australia and New Zealand). South Asian patients were the most vulnerable to under-screening, and decreasing patient income quintile was consistently associated with lower likelihood of screening, although less so for immigrant patients. This study highlights certain physician characteristics that are associated with cancer screening for eligible patients, including immigrant patients, and that should be considered when designing physician-targeted interventions. We have also highlighted an ethnic community, South Asians, which requires particular attention, both among its patients and its primary care providers. Future research should further explore the reasons for these findings.

## Introduction

Research has demonstrated that immigrants to Canada are under-screened for breast, cervical, and colorectal cancer (CRC), including in Ontario, Canada's most populous province 1–7. This inequality holds despite the known benefits of cancer screening and despite the three well-established cancer screening programs that exist in the province 8. The barriers to screening for immigrant groups are numerous and complex, and include patient-level factors, such as competing interests at physician visits, fatalistic views and lack of understanding of the need for preventive care, system-level factors such as lack of accommodation of different patient languages or cultural preferences, and physician-level factors 9–14.

Physicians, particularly primary care physicians, play a very important role in preventive care in general, and can serve as both facilitators and impeders to cancer screening. Evidence shows that physician recommendation can be a strong motivator for people to get screened, and correspondingly that lack of recommendation is a notable barrier 9,15–17. Physician-level barriers to screening for immigrants, such as lack of time to discuss screening, failure to tailor messages in a culturally appropriate manner, lack of physician knowledge about screening guidelines, and patient difficulties with accessing appointments, are therefore of utmost importance and need to be addressed in order to see meaningful increases in screening rates for immigrants and other disadvantaged groups 9,18–20.

Addressing these and other physician-level barriers will likely require targeted physician interventions. Such focused interventions would in turn benefit from knowledge of which physicians are most likely to have low (and high) screening rates for their patients, including their immigrant patients. For example, there is evidence to suggest that international medical graduates (IMGs) may be less likely to screen their patients for cancer than US- or Canadian-trained physicians, and that having a physician of the same ethnicity is associated with lower rates of cervical cancer screening 3,21,22. Therefore, the objective of this population-based retrospective cohort study was to inform physician-targeted screening interventions by identifying the characteristics of primary care physicians in Ontario that are associated with cancer screening for their eligible patients, for their eligible immigrant patients, and for IMG physicians, for their eligible immigrant patients from the same region of the world.

## Methods

### Data sources

We accessed several databases for this study through a comprehensive research agreement with Ontario's Ministry of Health and Long-Term Care. The databases were linked anonymously using unique identifiers. The Citizenship and Immigration Canada (CIC) database consists of detailed demographic information on Ontario's landed immigrants, recorded on the date of issue of the landing visa, and spans landing dates from 1985 to 2010. The Registered Persons Database includes the age, sex, and address of all Ontario residents who are eligible for the universal Ontario Health Insurance Plan. The Ontario Physicians' Claims Database contains diagnostic and procedural codes claimed by physicians in the province. The Ontario Cancer Registry records all Ontario residents who have been newly diagnosed with cancer or who have died of cancer. The Canadian Institute of Health Information Discharge Abstract Database contains administrative, demographic, and clinical data for inpatient hospital discharges. The Client Agency Program Enrollment database consists of all Ontarians who are enrolled in a primary health care patient enrollment model (PEM) and the Corporate Physicians' Database records which family physicians participate in these enrollment models. The Institute for Clinical Evaluative Sciences' Physicians' Database records demographic and specialty information about Ontario's physicians in active practice. The research protocol was approved by the Research Ethics Board at Sunnybrook Health Sciences Centre.

### Study population: physicians

As more than 90% of immigrants settle in metropolitan areas and as the majority of the province's population (74%) lives in metropolitan areas, we limited the study to primary care physicians who were in active practice in Ontario and whose primary practice site was in a metropolitan area 23,24. A metropolitan area is defined as a geographic area with an urban core whose population is at least 100,000 based on the Canadian Census. Physicians were also required to have been in independent practice for at least 3 years as of 31 December 2010. This date was chosen to correspond with the last date of available administrative data. Primary care physicians who had focused their practices (e.g., psychotherapy, sports medicine) and primary care physicians with less than 100 patients were excluded from the study cohort.

### Study population: patients

Patients were eligible for inclusion if they were alive as of 31 December 2010 and if they were either formally or virtually rostered to a study physician. Ontario has several kinds of primary care PEMs that allow patients to voluntarily formally roster with their primary care provider 25. Being virtually rostered refers to being assigned to a primary care physician, based on pattern of care, despite the patient not being formally rostered. Assignment is based on the family physician that has billed the largest dollar amount of services for that patient in the previous 2 years 26. This approach has been found to be accurate with 85% of patients appropriately virtually rostered to a family physician 26.

Included patients further had to be eligible for at least one of the cancer screening types. Female patients eligible for breast cancer screening were 50 to 74 years of age for all of 1 January 2009 to 31 December 2010 and had no prior diagnosis of invasive breast cancer. Female patients eligible for cervical cancer screening were 21 to 69 years of age for all of 1 January 2008 to 31 December 2010 and had no prior hysterectomy or prior diagnosis of invasive cervical cancer, endometrial cancer, or ovarian cancer. Patients considered eligible for CRC screening were 50–74 years of age for all of 1 January 2009 to 31 December 2010 and had no prior diagnoses of invasive CRC or inflammatory bowel disease. Ages were defined based on provincial screening guidelines 8.

### Study outcomes

Appropriate breast cancer screening was defined as at least one mammogram in the previous 2 years, appropriate cervical cancer screening was defined as at least one Pap test in the previous 3 years, and appropriate CRC screening was defined as at least one fecal occult blood test in the previous 2 years or at least one colonoscopy or barium enema in the previous 10 years. Time periods were defined based on provincial guidelines 8.

For each included primary care physician, study outcomes were determined for all of their screen-eligible patients, for all of their screen-eligible immigrant patients, and for IMG primary care physicians, for all of their eligible immigrant patients who were from the same region of the world. Physicians' world region of origin was defined based on medical school location and patients' world region of origin was defined based on country of birth. Countries were classified into world region based on a modification of the World Bank classification system 3,27.

### Data analysis

We used multivariable generalized estimating equation models to account for clustering by physician. We used logistic regression with a binary patient-level outcome for each cancer screening type: appropriately screened or not. Patient characteristics investigated were age, gender (for CRC screening), neighborhood income quintile based on the patient's postal code and Census data, immigrant status, and region of origin if the patient was an immigrant. Physician characteristics investigated were age, gender, years since medical school graduation, years in independent practice in Ontario, whether they were an IMG, world region of medical school if they were an IMG, and their patient panel size. Ontario's various PEMs have payment models that range from primarily fee-for-service to primarily capitation, and that financially incentivize cancer screening. Some capitation-based PEMs also are provided with funding to create interdisciplinary teams 25. Therefore, we considered if physicians participated in a PEM and, if so, what type. All variables were defined on 31 December 2010. Participants with missing responses were excluded. The analyses were performed using SAS version 9.3 (Cary, NC).

## Results

There were 6303 physicians included in the study (Table1), with a total of 9,344,636 patients in their practices. Of these, the breast screening cohort consisted of 1,156,627 patients, the cervical screening cohort had 2,730,380 patients, and the CRC screening cohort consisted of 2,260,569 patients (Table2). South Asia and East Asia were the most common regions of origin for immigrant patients. Table1 describes the physicians' demographic characteristics. Just fewer than 40% were female, and 26.1% were IMGs. Male physicians and IMG physicians tended to be slightly older, and accordingly to have graduated from medical school less recently, than their counterparts. The IMGs were least likely to participate in a PEM, and most likely to participate in a primarily fee-for-service-based PEM. Female physicians had somewhat smaller practice sizes than their male counterparts, and IMGs had a higher proportion of immigrants in their practices than Canadian graduates.

**Table 1:** Demographic characteristics of the 6303 primary care physicians included in the study

Variable	Value	Total (*N* = 6303)	Female (*n* = 2513)	Male (*n* = 3790)	Canadian medical graduate (*n* = 4656)	International medical graduate (*n* = 1647)
Sex	Female	2513 (39.9%)			1890 (40.6%)	623 (37.8%)
	Male	3790 (60.1%)			2765 (59.4%)	1024 (62.2%)
Type of medical graduate	Canadian	4656 (73.9%)	1890 (75.2%)	2766 (73.0%)		
	International	1647 (26.1%)	623 (24.8%)	1024 (27.0%)		
World region of medical school	Caribbean/Latin America					76 (4.6%)
	East Asia					160 (9.7%)
	Eastern Europe					180 (10.9%)
	Middle East/North Africa					264 (16.0%)
	South Asia					331 (20.1%)
	Sub-Saharan Africa					131 (8.0%)
	USA/Australia/New Zealand					57 (3.5%)
	Western Europe					448 (27.2%)
Age as of Dec 31, 2010	Mean ± SD	52.85 ± 10.95	48.80 ± 9.64	55.53 ± 10.93	51.44 ± 10.36	56.83 ± 11.57
	Median (IQR)	53.0 (45.0–60.0)	49.0 (41.0–55.0)	56.0 (47.0–63.0)	51.0 (44.0–59.0)	57.0 (48.0–66.0)
Years since medical school graduation	Mean ± SD	26.41 ± 11.28	22.40 ± 10.08	29.07 ± 11.25	24.69 ± 10.66	31.28 ± 11.58
	Median (IQR)	26.0 (18.0–34.0)	22.0 (15.0–29.0)	29.0 (21.0–37.0)	24.0 (17.0–32.0)	31.0 (23.0–40.0)
Years in independent practice in Ontario	Mean ± SD	18.93 ± 8.12	16.36 ± 7.89	20.63 ± 7.82	19.51 ± 7.42	17.28 ± 9.65
	Median (IQR)	20.34 (12.54–23.58)	18.52 (8.90–21.76)	21.01 (17.51–24.92)	20.44 (14.55–23.52)	19.89 (7.37–23.75)
Type of patient enrollment model	Team-based	1053 (16.7%)	492 (19.6%)	561 (14.8%)	914 (19.6%)	139 (8.4%)
	Capitation-based	1380 (21.9%)	585 (23.3%)	795 (21.0%)	1166 (25.0%)	214 (13.0%)
	Fee-for-service	2794 (44.3%)	1093 (43.5%)	1701 (44.9%)	1853 (39.8%)	941 (57.1%)
	None	1059 (16.8%)	341 (13.6%)	718 (18.9%)	706 (15.2%)	353 (21.4%)
Patient panel size	Mean ± SD	1482.47 ± 840.31	1280.49 ± 708.85	1616.40 ± 892.36	1421.34 ± 800.93	1655.29 ± 921.33
	Median (IQR)	1400.0 (876.0–1971.0)	1213.0 (803.0–1654.0)	1567.0 (979.0–2160.0)	1343.50 (848.50–1895.0)	1578.0 (997.0–2221.0)
Proportion of patients who are immigrants	Mean ± SD	0.16 ± 0.17	0.16 ± 0.16	0.17 ± 0.17	0.12 ± 0.13	0.28 ± 0.20
	Median (IQR)	0.10 (0.04–0.23)	0.09 (0.04–0.21)	0.10 (0.04–0.25)	0.07 (0.03–0.16)	0.25 (0.10–0.45)
Proportion of patients who are immigrants from the same region	Mean ± SD					0.14 ± 0.19
	Median (IQR)					0.04 (0.01–0.22)

**Table 2:** Patients included in each screening cohort by region of origin

Region	Breast		Cervical		Colorectal	
	* N*	%	* N*	%	* N*	%
All regions	1,156,627	100.0	2,730,380	100.0	2,260,569	100.0
Caribbean and Latin America	25,629	2.2	89,096	3.3	46,775	2.1
Canada	987,779	85.4	2,111,371	77.3	1,928,768	85.3
East Asia and Pacific	51,243	4.4	178,030	6.5	93,784	4.2
Eastern Europe and Central Asia	26,493	2.3	88,265	3.2	51,579	2.3
Middle East and North Africa	11,821	1.0	46,994	1.7	25,865	1.1
South Asia	36,991	3.2	142,459	5.2	76,683	3.4
Sub-Saharan Africa	6710	0.6	35,253	1.3	15,480	0.7
USA, Australia, and New Zealand	2532	0.2	9469	0.4	4915	0.2
Western Europe	7429	0.6	29,443	1.1	16,720	0.7

**Figure 1:**
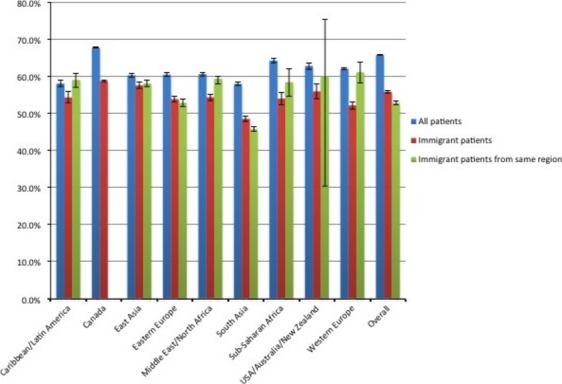
Breast cancer screening rates by region of physician medical school, for all eligible patients, all eligible immigrant patients and, for international medical graduate (IMG) physicians, all eligible immigrant patients from the same region of the world.

**Figure 2:**
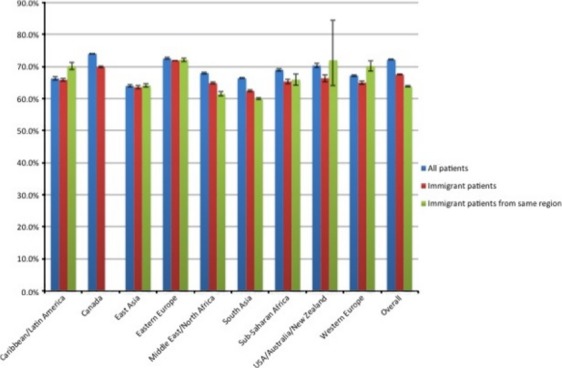
Cervical cancer screening rates by region of physician medical school, for all eligible patients, all eligible immigrant patients and, for international medical graduate (IMG) physicians, all eligible immigrant patients from the same region of the world.

**Figure 3:**
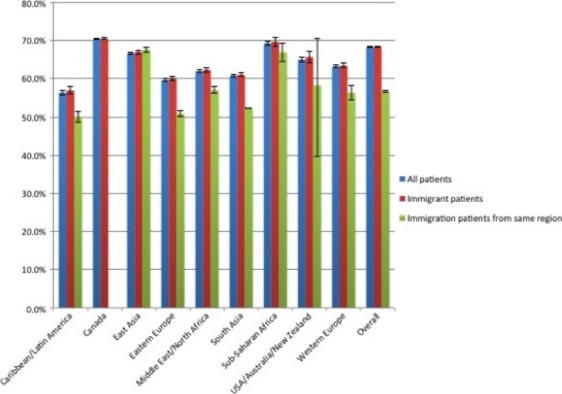
Colorectal cancer screening rates by region of physician medical school, for all eligible patients, all eligible immigrant patients and, for international medical graduate (IMG) physicians, all eligible immigrant patients from the same region of the world.

Figures3 describe the screening rates for each cancer screening type for all eligible patients, for all eligible immigrant patients, and for IMG physicians, for all eligible immigrant patients from the same region of the world. For breast cancer (Fig.1), Canadian graduates had the highest proportion of patients screened, both overall and for foreign-born patients, although the latter group had lower screening rates. South Asian-trained physicians had the lowest screening rates, particularly for immigrant patients who were also South Asian, whereas other IMG physicians tended to have similar or higher rates of screening for patients from the same world region. For cervical cancer (Fig.2), Canadian graduates again had the highest proportion of patients screened, although Eastern European-trained physicians had the highest proportion of immigrant patients screened. South Asian and Middle Eastern/North African-trained physicians had lower rates of screening for immigrant women, and even lower rates for women from the same region of the world. In contrast, women from the Caribbean/Latin America and from Western Europe seemed to benefit from seeing a physician trained in the same region of the world when it came to cervical cancer screening. For CRC screening (Fig.3), Caribbean/Latin American-trained physicians had the lowest screening rates. Across all regions of medical school graduation, screening rates tended to be quite similar between all patients and all immigrant patients, but to be noticeably lower for patients who were from the same region of the world as their primary care physician.

Results of multivariable analyses are presented in Table3, with statistically significant variables highlighted. Several variables were significantly associated with screening across all patient groups and cancer types, namely, patient income quintile, physician sex, and participation in a PEM. Adjusted odds ratios for income quintile tended to be higher for all patients than for the immigrant patient group. When looking at all screen-eligible patients, patient region of origin and the physician's years in independent practice and region of medical school were also associated with screening. When compared to nonimmigrant patients, patients from the Caribbean/Latin America were the only immigrant group to not consistently have lower odds of screening across cancer types. As well, South Asian patients consistently had the lowest adjusted odds ratios. Physicians who had graduated from medical schools in the Caribbean/Latin America, the Middle East/North Africa, South Asia, and Western Europe had consistently lower odds of screening their eligible patients across cancer types when compared to Canadian medical school graduates. Patient sex was associated with CRC screening with female patients having higher odds of screening. When looking at immigrant patients only, patient region of origin was again significantly associated with screening, and patient sex was again associated with CRC screening. The physician's years in independent practice was significant for breast and CRC screening. For immigrant patients who were rostered to physicians from the same region of the world, the particular region was only significant for South Asian women for cervical cancer screening (adjusted odds ratio 0.56 [95% confidence interval 0.32–0.98]).

**Table 3:** Adjusted odds ratios and 95% confidence intervals derived from multivariate analyses for breast, cervical, and colorectal cancer screening for all eligible patients, all eligible immigrant patients, and all eligible immigrant patients seeing a primary care physician from the same region of the world

Variable	Breast cancer screening	Cervical cancer screening	Colorectal cancer screening
*All patients*			
Patient sex			
Female			1.12 (1.11, 1.14)*
Male			1.0
Patient age (1 year older)	1.00 (1.00, 1.00)	0.98 (0.98, 0.98)*	1.02 (1.02, 1.03)*
Patient income quintile			
Q1 (lowest)	1.0	1.0	1.0
Q2	1.21 (1.20, 1.23)*	1.17 (1.15, 1.18)*	1.15 (1.14, 1.17)*
Q3	1.32 (1.30, 1.34)*	1.34 (1.32, 1.36)*	1.26 (1.23, 1.28)*
Q4	1.43 (1.41, 1.46)*	1.50 (1.48, 1.52)*	1.38 (1.35, 1.41)*
Q5 (highest)	1.54 (1.51, 1.57)*	1.62 (1.59, 1.64)*	1.59 (1.55, 1.62)*
Patient region of origin			
Caribbean and Latin America	1.04 (1.00, 1.08)	1.29 (1.25, 1.33)*	0.96 (0.91, 1.01)
Canada	1.0	1.0	1.0
East Asia	0.80 (0.76, 0.83)*	0.91 (0.87, 0.94)*	0.99 (0.93, 1.05)
Eastern Europe	0.61 (0.57, 0.64)*	0.94 (0.90, 0.99)*	0.64 (0.59, 0.69)*
Middle East and North Africa	0.82 (0.75, 0.91)*	0.68 (0.64, 0.72)*	0.73 (0.65, 0.82)*
South Asia	0.51 (0.48, 0.54)*	0.61 (0.59, 0.64)*	0.61 (0.56, 0.65)*
Sub-Saharan Africa	0.71 (0.66, 0.77)*	0.83 (0.79, 0.88)*	0.79 (0.74, 0.84)*
USA, Australia, and New Zealand	0.69 (0.63, 0.75)*	0.88 (0.83, 0.92)*	0.87 (0.82, 0.93)*
Western Europe	0.86 (0.81, 0.92)*	0.96 (0.92, 1.00)	0.78 (0.72, 0.84)*
Physician sex			
Female	1.43 (1.39, 1.48)*	1.97 (1.90, 2.05)*	1.37 (1.31, 1.44)*
Male	1.0	1.0	1.0
Physician age (1 year older)	0.99 (0.99, 1.00)	0.99 (0.99, 1.00)	0.99 (0.98, 1.00)
Years since medical school graduation (additional 1 year after graduation)	1.00 (1.00, 1.01)	1.00 (1.00, 1.01)	1.00 (0.99, 1.01)
Years in independent practice in Ontario			
More than 20 years	1.12 (1.06, 1.19)*	1.13 (1.06, 1.20)*	1.32 (1.21, 1.45)*
15–19 years	1.11 (1.05, 1.17)*	1.10 (1.04, 1.16)*	1.27 (1.17, 1.38)*
10–14 years	1.08 (1.02, 1.14)*	1.03 (0.97, 1.10)	1.13 (1.04, 1.23)*
Less than 10 years	1.0	1.0	1.0
Patient panel size (100 additional patients)	1.00 (0.99, 1.00)	1.00 (0.99, 1.00)	1.00 (1.00, 1.00)
Region of physician medical school			
Caribbean and Latin America	0.84 (0.74, 0.96)*	0.87 (0.78, 0.98)*	0.72 (0.59, 0.88)*
Canada	1.0	1.0	1.0
East Asia	0.96 (0.86, 1.08)	0.83 (0.76, 0.91)*	1.14 (0.99, 1.32)
Eastern Europe	0.91 (0.83, 0.99)*	0.97 (0.88, 1.07)	0.85 (0.74, 0.98)*
Middle East and North Africa	0.84 (0.79, 0.91)*	0.82 (0.76, 0.88)*	0.88 (0.78, 0.98)*
South Asia	0.85 (0.80, 0.91)*	0.80 (0.75, 0.87)*	0.83 (0.74, 0.93)*
Sub-Saharan Africa	0.96 (0.88, 1.05)	0.93 (0.84, 1.02)	1.08 (0.94, 1.25)
USA, Australia, and New Zealand	0.93 (0.79, 1.10)	0.98 (0.83, 1.16)	0.95 (0.75, 1.21)
Western Europe	0.90 (0.85, 0.95)*	0.88 (0.83, 0.93)*	0.88 (0.81, 0.96)*
Patient enrollment model type			
Team-based	1.93 (1.81, 2.06)*	1.73 (1.60, 1.86)*	2.30 (2.12, 2.50)*
Capitation-based	1.73 (1.63, 1.84)*	1.78 (1.67, 1.89)*	2.50 (2.30, 2.71)*
Fee-for-service-based	1.61 (1.52, 1.72)*	1.67 (1.57, 1.78)*	2.32 (2.15, 2.50)*
None	1.0	1.0	1.0
*Immigrant patients*			
Patient sex			
Female			1.05 (1.03, 1.07)*
Male			1.0
Patient age (1 year older)	0.97 (0.96, 0.97)*	0.98 (0.98, 0.99)*	1.01 (1.00, 1.01)
Patient income quintile			
Q1 (lowest)	1.0	1.0	1.0
Q2	1.12 (1.09, 1.16)*	1.11 (1.09, 1.13)*	1.12 (1.08, 1.15)*
Q3	1.17 (1.13, 1.21)*	1.20 (1.18, 1.23)*	1.19 (1.15, 1.23)*
Q4	1.25 (1.20, 1.29)*	1.33 (1.29, 1.36)*	1.27 (1.23, 1.32)*
Q5 (highest)	1.19 (1.14, 1.25)*	1.30 (1.27, 1.34)*	1.32 (1.27, 1.38)*
Patient region of origin			
Caribbean and Latin America	1.43 (1.30, 1.58)*	1.34 (1.26, 1.42)*	1.05 (0.97, 1.15)
East Asia	1.11 (1.00, 1.23)*	0.94 (0.89, 1.01)	1.10 (1.00, 1.20)*
Eastern Europe	0.82 (0.74, 0.91)*	1.00 (0.94, 1.07)	0.72 (0.65, 0.79)*
Middle East and North Africa	1.17 (1.03, 1.32)*	0.72 (0.67, 0.77)*	0.81 (0.72, 0.91)*
South Asia	0.71 (0.64, 0.78)*	0.62 (0.58, 0.66)*	0.66 (0.61, 0.73)*
Sub-Saharan Africa	0.97 (0.86, 1.09)	0.86 (0.81, 0.92)*	0.85 (0.78, 0.93)*
USA, Australia, and New Zealand	1.0	1.0	1.0
Western Europe	1.24 (1.12, 1.38)*	1.06 (1.00, 1.13)	0.87 (0.79, 0.96)*
Physician sex			
Female	1.43 (1.35, 1.53)*	1.80 (1.70, 1.91)*	1.28 (1.17, 1.39)*
Male	1.0	1.0	1.0
Physician age (1 year older)	0.99 (0.98, 1.00)	0.99 (0.99, 1.00)	0.98 (0.97, 1.00)*
Years since medical school graduation (additional 1 year after graduation)	1.01 (0.99, 1.02)	1.00 (0.99, 1.01)	1.01 (0.99, 1.02)
Years in independent practice in Ontario			
More than 20 years	1.13 (1.02, 1.25)*	1.02 (0.93, 1.12)	1.26 (1.08, 1.46)*
15–19 years	1.19 (1.08, 1.32)*	1.04 (0.96, 1.13)	1.25 (1.09, 1.43)*
10–14 years	1.13 (1.01, 1.27)*	1.02 (0.93, 1.11)	1.10 (0.95, 1.26)
Less than 10 years	1.0	1.0	1.0
Patient panel size (100 additional patients)	1.00 (0.99, 1.00)	1.00 (1.00, 1.00)	1.00 (1.00, 1.01)
Region of physician medical school			
Caribbean and Latin America	0.87 (0.75, 1.01)	0.87 (0.77, 0.98)*	0.67 (0.52, 0.85)*
Canada	1.0	1.0	1.0
East Asia	1.02 (0.88, 1.18)	0.86 (0.78, 0.95)*	1.14 (0.95, 1.36)
Eastern Europe	1.00 (0.88, 1.14)	1.02 (0.90, 1.15)	0.82 (0.68, 1.00)
Middle East and North Africa	0.90 (0.80, 1.01)	0.85 (0.77, 0.94)*	0.88 (0.74, 1.05)
South Asia	0.92 (0.83, 1.02)	0.87 (0.79, 0.95)*	0.87 (0.74, 1.02)
Sub-Saharan Africa	0.93 (0.80, 1.09)	0.91 (0.80, 1.03)	0.98 (0.80, 1.21)
USA, Australia, and New Zealand	0.99 (0.80, 1.23)	0.95 (0.78, 1.17)	1.02 (0.76, 1.38)
Western Europe	0.90 (0.82, 1.00)	0.90 (0.82, 0.98)*	0.87 (0.75, 1.01)
Patient enrollment model type			
Team-based	1.78 (1.60, 1.98)*	1.50 (1.34, 1.67)*	2.51 (2.19, 2.87)*
Capitation-based	1.61 (1.47, 1.77)*	1.57 (1.44, 1.72)*	2.74 (2.42, 3.10)*
Fee-for-service-based	1.60 (1.47, 1.73)*	1.56 (1.43, 1.71)*	2.44 (2.19, 2.72)*
None	1.0	1.0	1.0
*Immigrant patients rostered to physician from same world region*			
Patient sex			
Female			1.00 (0.97, 1.04)
Male			1.0
Patient age (1 year older)	0.96 (0.96, 0.97)*	0.98 (0.98, 0.99)*	1.00 (1.00, 1.01)
Patient income quintile			
Q1 (lowest)	1.0	1.0	1.0
Q2	1.11 (1.05, 1.18)*	1.08 (1.03, 1.13)*	1.12 (1.06, 1.19)*
Q3	1.15 (1.08, 1.23)*	1.17 (1.10, 1.23)*	1.19 (1.11, 1.27)*
Q4	1.30 (1.21, 1.39)*	1.27 (1.20, 1.34)*	1.30 (1.21, 1.39)*
Q5 (highest)	1.30 (1.16, 1.39)*	1.25 (1.17, 1.34)*	1.31 (1.20, 1.44)*
Physician sex			
Female	1.25 (1.10, 1.43)*	1.71 (1.52, 1.92)*	1.14 (0.96, 1.35)
Male	1.0	1.0	1.0
Physician age (1 year older)	0.99 (0.97, 1.02)	0.99 (0.97, 1.02)	0.97 (0.94, 1.01)
Years since medical school graduation (additional 1 year after graduation)	1.00 (0.98, 1.02)	1.00 (0.97, 1.02)	1.01 (0.98, 1.05)
Years in independent practice in Ontario			
More than 20 years	0.99 (0.81, 1.22)	0.96 (0.80, 1.14)	1.16 (0.88, 1.53)
15–19 years	1.21 (1.00, 1.47)	1.05 (0.89, 1.23)	1.16 (0.89, 1.51)
10–14 years	1.19 (0.96, 1.49)	1.15 (0.96, 1.37)	1.16 (0.88, 1.52)
Less than 10 years	1.0	1.0	1.0
Patient panel size (100 additional patients)	1.00 (0.99, 1.00)	1.00 (0.99, 1.01)	1.00 (0.99, 1.01)
Region of physician medical school			
Caribbean and Latin America	1.38 (0.58, 3.28)	1.25 (0.70, 2.21)	0.90 (0.38, 2.15)
East Asia	1.18 (0.50, 2.80)	0.85 (0.48, 1.51)	1.59 (0.68, 3.72)
Eastern Europe	0.89 (0.38, 2.11)	1.08 (0.61, 1.92)	0.80 (0.34, 1.87)
Middle East and North Africa	1.17 (0.49, 2.82)	0.60 (0.34, 1.07)	0.93 (0.39, 2.25)
South Asia	0.70 (0.30, 1.66)	0.56 (0.32, 0.98)*	0.75 (0.32, 1.74)
Sub-Saharan Africa	1.15 (0.46, 2.86)	0.90 (0.49, 1.66)	1.39 (0.57, 3.39)
USA, Australia, and New Zealand	1.0	1.0	1.0
Western Europe	1.24 (0.51, 3.03)	1.04 (0.58, 1.87)	0.94 (0.39, 2.27)
Patient enrollment model type			
Team-based	1.44 (1.06, 1.94)*	1.43 (1.12, 1.83)*	2.10 (1.37, 3.21)*
Capitation-based	1.37 (1.10, 1.69)*	1.32 (1.09, 1.60)*	2.59 (1.89, 3.54)*
Fee-for-service-based	1.33 (1.14, 1.54)*	1.35 (1.14, 1.60)*	2.29 (1.89, 3.54)*
None	1.0	1.0	1.0

* Statistically significant result.

## Discussion

In Ontario's metropolitan areas, we have demonstrated that seeing a physician who is male, who does not participate in any type of PEM, and/or who has been in independent practice for a shorter period of time tends to be associated with a decreased likelihood of screening patients for cancer, including immigrant patients. In multivariable analyses, physicians who attended medical schools in certain regions of the world, specifically the Caribbean/Latin America, the Middle East/North Africa, South Asia, and Western Europe were less likely to screen their patients than Canadian graduates. As well, South Asian IMGs were significantly less likely to screen South Asian women for cervical cancer than IMGs from other regions of the world who were seeing region-congruent patients. Our results confirm previous findings that immigrants are under-screened for cancer in Ontario, with Caribbean/Latin American patients being a notable exception. South Asian patients were the most vulnerable to under-screening in general, and decreasing patient income quintile was consistently associated with lower likelihood of screening, although less so for immigrant patients. Male patients, both immigrant and nonimmigrant, were generally less likely to participate in CRC screening.

By highlighting particular physician characteristics associated with cancer screening, our study findings have several implications regarding on whom to focus physician-targeted interventions and education campaigns. Our results suggest that such interventions may be most effective by starting with males, non-PEM physicians, those who have been in independent practice a shorter period of time, and IMGs from certain world regions. In particular, Ontario's PEMs provide financial incentives for cancer screening, and our findings suggest that ways need to be found to encourage those physicians who are not currently participating in PEMs to do so. Identifying ways to intervene with particular physician demographic groups without appearing to be discriminatory will be essential to success. Partnering with existing physician associations that are based on ethnic origin or focusing on geographic regions where physicians of certain ethnicities cluster may hold some promise in this regard.

At the same time that targeted interventions are being explored, further exploration of the reasons for our findings is required. For example, the reason why patients of male physicians are less likely to be screened needs to be further examined and addressed. The intimate procedure of the Pap test may make many patients, especially from certain cultural groups, more comfortable with having a female physician perform the test and similarly, female physicians may be more comfortable performing the test than male physicians 28. However, this would not be expected to apply to breast and CRC screening which are not directly performed by the primary care physician. Similarly, the reason why IMGs from certain regions are less likely to perform screening needs to be explored. Although there may be differences in medical school curriculums around the world, with some schools putting less emphasis on prevention, many IMGs will have completed their residency training in Ontario 29. Certainly the importance of cultural and religious factors needs to be considered.

Both South Asian-trained physicians and South Asian-born patients were highlighted in our study findings, with the former being especially likely to under-screen their patients, in particular their South Asian female patients for cervical cancer, and with the latter being especially vulnerable to under-screening. These findings correlate with our previous research and with other literature 2,3,10,30. In our previous work, we found that patients' fears, values and beliefs, and physician lack of knowledge were among the reasons for under-screening among South Asians in Ontario 9. Accordingly, we are involved in both patient-level and physician-level interventions that are underway in the province to address cancer screening for South Asian patients and primary care physicians. As South Asians are one of the province's largest minority groups and considering that South Asian-trained IMGs made up of 20% of IMGs in this study, these inequalities will affect cancer screening at the population level and need to continue to be examined 31.

Few Canadian studies have explored physician characteristics associated with cancer screening. Dahrouge et al. found that Ontario practices with at least one female primary care provider, with fewer than 1600 patients per full-time provider, and with an electronic reminder system were more likely to provide evidence-based preventive health care, including cancer screening 32. Research by one of the authors (A. L.) suggested that not being in a PEM, having a male primary care provider, and having a primary care provider from the same region of the world were associated with lower rates of cervical cancer screening in Ontario for immigrant women 3. Tu et al. found that having a Chinese male provider was negatively associated with breast and cervical cancer screening in Vancouver, British Columbia for Chinese women 33. In Decker et al.'s 2009 study conducted in the province of Manitoba, primary care providers who were rural, male, or IMGs were less likely to provide cervical cancer screening 34. The current study adds to the literature due to examining all three evidence-based cancer screening types and due to the level of detail of physician demographics that we explored. The positive benefit of female providers on preventive care that we found has been well noted in the international literature 28,35–38.

This study has several limitations. First, because of our use of secondary administrative data that were not explicitly collected for research purposes, it is not possible to know why patients were or were not screened. Some patients may not have been offered screening, and others may have been offered screening but refused. For example, male physicians may have offered female patients Pap testing and been refused. As mentioned, the reasons for screening differences we highlighted needs to be explored. Factors such as access to translation services for non-English speakers should be assessed in future work. Second, the CIC database that was used to identify immigrant patients is not complete. It does not include immigrants who came to Canada before 1985 or those who declared at the time of landing that they would move to another Canadian province but ended up in Ontario. Therefore, some immigrant patients may have been incorrectly assigned as being Canadian-born. Third, a physician's medical school region might not always reflect their region of origin. For example, there are many medical schools in the Caribbean that cater primarily to North American students 39. Therefore, we may have incorrectly assigned some patients as being from the same region of the world as their physicians. However, there were no other data available that would allow determination of a physician's region of origin.

In this study, we have highlighted certain physician characteristics that are associated with breast, cervical, and CRC screening for screen-eligible patients, including immigrant patients, and for IMG physicians, for patients from the same region of the world. These characteristics should be considered when considering physician-targeted interventions in Ontario. We have also highlighted an ethnic community that requires particular attention, both among its patients and its primary care providers. Future research, ideally including primary data collection and qualitative methods, should further explore the reasons for our findings. Participation in PEMs has steadily increased since the study period and it would also be important to explore if physician screening rates increased accordingly. Exploring screening and prevention practices for other chronic conditions may also prove informative. Policymakers and public health practitioners should consider these results when designing screening interventions.
